# Clinical trials in a remote Aboriginal setting: lessons from the BOABS smoking cessation study

**DOI:** 10.1186/1471-2458-14-579

**Published:** 2014-06-10

**Authors:** Julia V Marley, Tracey Kitaura, David Atkinson, Sue Metcalf, Graeme P Maguire, Dennis Gray

**Affiliations:** 1 The Rural Clinical School of Western Australia, The University of Western Australia, 12 Napier Terrace, PO Box 1377, Broome, WA 6725, Australia; 2 Kimberley Aboriginal Medical Services Council, 12 Napier Terrace, PO Box 1377, Broome, Western Australia 6725, Australia; 3 Derby Aboriginal Health Service, 1 Stanley Street, PO Box 1155, Derby, Western Australia 6728, Australia; 4 School of Medicine and Dentistry, James Cook University, Cairns, Queensland 4870, Australia; 5 Baker IDI, Alice Springs, Northern Territory 0871, Australia; 6 National Drug Institute, Curtin University, GPO Box U1987, Perth, Western Australia 6845, Australia

**Keywords:** Indigenous, Aboriginal, Torres Strait Islander, Smoking cessation, Be Our Ally Beat Smoking (BOABS) study, Qualitative, Randomised controlled trial

## Abstract

**Background:**

There is limited evidence regarding the best approaches to helping Indigenous Australians to stop smoking. The composite analysis of the only two smoking cessation randomised controlled trials (RCTs) investigating this suggests that one-on-one extra support delivered by and provided to Indigenous Australians in a primary health care setting appears to be more effective than usual care in encouraging smoking cessation. This paper describes the lessons learnt from one of these studies, the Be Our Ally Beat Smoking (BOABS) Study, and how to develop and implement an integrated smoking cessation program.

**Methods:**

Qualitative study using data collected from multiple documentary sources related to the BOABS Study. As the project neared completion the research team participated in four workshops to review and conduct thematic analyses of these documents.

**Results:**

Challenges we encountered during the relatively complex BOABS Study included recruiting sufficient number of participants; managing the project in two distant locations and ensuring high quality work across both sites; providing appropriate training and support to Aboriginal researchers; significant staff absences, staff shortages and high workforce turnover; determining where and how the project fitted in the clinics and consequent siloing of the Aboriginal researchers relating to the requirements of RCTs; resistance to change, and maintaining organisational commitment and priority for the project. The results of this study also demonstrated the importance of local Aboriginal ownership, commitment, participation and control. This included knowledge of local communities, the flexibility to adapt interventions to local settings and circumstances, and taking sufficient time to allow this to occur.

**Conclusions:**

The keys to the success of the BOABS Study were local development, ownership and participation, worker professional development and support, and operating within a framework of cultural safety. There were difficulties associated with the BOABS Study being an RCT, and many of these are shared with stand-alone programs. Interventions targeted at particular health problems are best integrated with usual primary health care. Research to investigate complex interventions in Indigenous health should not be limited to randomised clinical trials and funding needs to reflect the additional, but necessary, cost of providing for local control of planning and implementation.

## Background

In 2008, the age-standardised prevalence of current smoking among Aboriginal and Torres Strait Islander peoples (Indigenous Australians) was more than double that among other Australians (49.8% compared with 20.5% of those aged 18 years and over) [1]. This contributes to higher rates of hospitalisation and death from tobacco-related conditions and self-reported ill-health and to the wider health disparity between Indigenous and non-Indigenous Australians [2-5]. For this reason, reducing the prevalence of smoking is important for everyday primary health care practice − particularly for services with significant Indigenous Australian clientele. However, evidence regarding the best approaches to assisting Aboriginal and Torres Strait Islander peoples to stop smoking is limited.

In 2004, Aboriginal Community Controlled Health Services (ACCHS) in the Kimberley region of northern Western Australia began to formally target reducing the prevalence of Indigenous smoking. This included provision of subsidised nicotine replacement therapy (NRT) in 2004, and the decision by the Kimberley Aboriginal Medical Services Council to implement a ‘smoke free’ workplace strategy in 2006. To address the issue of limited evidence regarding the best approaches to intervention a randomised controlled trial (RCT) was undertaken to evaluate an intensive Aboriginal led and supported smoking cessation intervention in comparison with standard care at two Kimberley ACCHSs − the Be Our Ally Beat Smoking (BOABS) Study [6,7].

Clients wanting to quit were randomly allocated to: either a control group which received usual care including advice from clinical staff, NRT and self-initiated follow-up; or an intensive care group which received usual care plus smoking cessation counselling at face-to-face visits from BOABS Aboriginal Researchers (ARs). Overall, the quit rate in the intensive support group was double that of the usual care group [7]. Unfortunately, due to recruiting difficulties, the study was under-powered and the results were not statistically significant. Nevertheless, when the results of the BOABS Study were pooled with those from the only other published study investigating the long-term benefit of personal support interventions in primary care [8] – a study with similar point estimates − the results suggest that one-on-one intensive intervention delivered by and provided to Aboriginal and Torres Strait Islander people in a primary health care setting is more effective than usual care in encouraging smoking cessation (rate ratio of 2.4; 95% CI 1.01 − 5.53) [7].

Integrated, culturally safe services provided by ACCHSs have been shown to be effective [9-12], nonetheless establishing and sustaining new stand-alone intervention programs in addition to individual client-based primary care in those settings can be extremely complex. Whilst new programs delivered by enthusiastic outsiders can provide short-term benefits to Indigenous Australians, once staff leave these programs often lose momentum [13]. Thus, recruiting, supporting, training and employing local Aboriginal and Torres Strait Islander people is one of the central elements in the provision of sustainable Indigenous health programs, especially in remote areas [14]. Such complexity also applies to research projects and cautionary reports have been published on the difficulties of conducting RCTs in ACCHSs settings [15,16]. Qualitative analysis of the processes involved in the BOABS Study provides a number of lessons for the establishment, implementation and integration of both intervention and research programs into standard primary health care practice. It is the aim of this paper to discuss some of the more salient lessons learnt from BOABS to inform future smoking cessation and health research activities in this setting.

## Methods

The lessons learnt component of the BOABS Study used participatory action research that involved all project staff and chief investigators (CIs). The CIs were aware that outcomes of interventions are crucially dependent upon the processes involved. Accordingly, a decision was made to use a qualitative approach to recording BOABS Study process data and a number of strategies were undertaken to collect them.

From the start of the BOABS Study, all project staff were required to maintain diaries in which were recorded routine activities, participants contacted and other project related information. Staff also wrote reports and personal ‘reflections’ , including: strategies for engaging with participants; barriers to recruitment and implementation; strategies to address barriers; and comments on the project more broadly. Recording of information in diaries was initially limited, but this was enhanced by ensuring closer involvement and regular engagement with the coordinating CI (JM). At workshops in 2010, the CIs refined guidelines for preparation of the diaries and reflections, and the coordinating CI provided day-to-day guidance and assistance to the ARs. The diaries and reflections were prepared as Microsoft Word (Microsoft, Seattle, USA) documents and regularly e-mailed to the coordinating CI to review and request clarification as required. These observations and notes form the basis of this paper and all project staff contributed to them.

The project manager/coordinating CI and the ARs had weekly teleconferences, regular local meetings, and meetings with the other CIs. Issues arising from the diaries and reflections, and other issues identified in the conduct of the project (e.g., slow recruitment rates) were discussed. Proceedings of meetings and actions were minuted and, along with the diaries and reflections, were kept in an electronic file maintained by the coordinating CI.

In 2010–11 several additional smoking cessation and healthy lifestyle positions were funded in the Kimberley through Council of Australian Governments (COAG) and Western Australian State Government Aboriginal health strategies. These tobacco action workers (5) and regional coordinators (2) were informally interviewed about their experiences in conducting tobacco cessation programs and the relevance this might have for the BOABS Study. Again, notes of these discussions were recorded and stored as Microsoft Word files.

**Figure 1:**
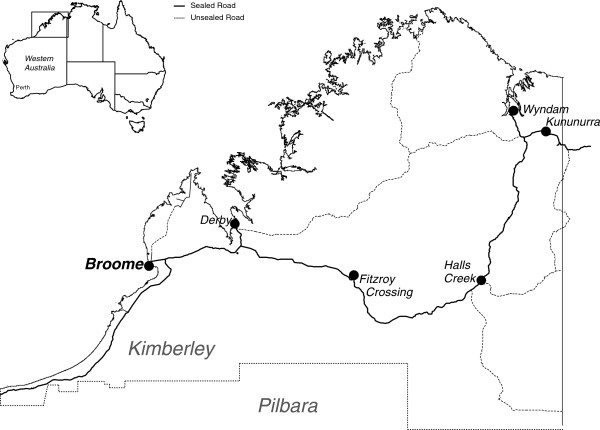
Sites where the Be Our Ally Beat Smoking (BOABS) Study was conducted, the Kimberley, Western Australia.

As the project neared completion the CIs and ARs participated in four workshops to review and conduct thematic analyses of these various documentary sources. The initial focus of the review of individual documents was to identify factors that facilitated or provided barriers to undertaking the project or ‘other’ issues, with segments of the text coded appropriately. As review of the individual documents continued, the initial coding categories were further divided and refined and a hierarchical coding scheme was developed based on group consensus.At least three members of the research team were involved in each workshop. The coding was an iterative process and the codes were revised in the light of discussion and analysis. That this analysis was undertaken in three remote primary health care services (see Figure 1) imposed its own constraints (e.g., availability of staff) and we endeavoured to undertake a process that was appropriate in this context. It was not possible to exclude individuals who wrote the notes, from the coding process, hence the importance we placed on reaching group consensus.

Once individual documents had been coded, they were amalgamated into a textual database using the tabular functions of Microsoft Word. Data segments – identifiable by document source and date – were sorted by code and the coding categories were again further refined. Concurrently, the content of the various coding categories was reviewed and important and recurring themes identified. Preliminary conclusions were developed and these conclusions were then tested using data from both within the database and the outcomes of the larger BOABS Study. The results of these analyses are presented below.

The number of participants from Derby and Kununurra who stopped smoking 12 months after enrolment was taken from the outcomes of the larger BOABS Study [7]. Differences in smoking cessation rates between Derby and Kununurra were compared using χ^2^ tests.

### Ethics approval

The BOABS Study was conducted within the framework of the National Health and Medical Research Council’s *Values And Ethics: Guidelines For Ethical Conduct In Aboriginal And Torres Strait Islander Health Research*[17]. The Study received approval from The University of Western Australia Human Research Ethics Committee and the Western Australian Aboriginal Health Information and Ethics Committee, and support from the Kimberley Aboriginal Health Planning Forum Kimberley Research Subcommittee.

## Results

Implementing the BOABS Study was complex, with particular issues being clearly defining the intervention, project management (including staff turnover), recruitment of participants, training and support of project staff and implementation.A key issue in conducting the BOABS Study as an RCT was recruitment of a sufficient number of participants to provide the statistical power to detect a real difference in cessation rates. Recognising this, we initially proposed to conduct the trial at two sites – the Broome Regional Aboriginal Medical Service (BRAMS) in Broome, and Derby Aboriginal Health Service (DAHS) in Derby. However, after funding was obtained, BRAMS informed the CIs that they were not in a position to participate, but fortunately the Ord Valley Aboriginal Health Service (OVAHS) in Kununurra agreed to do so. This meant the Study had to be managed from Broome across two sites that were 220 km (Derby) and 1044 km (Kununurra) distant (see Figure 1). Due to distance, Kununurra was more expensive and difficult to support and this had implications for the conduct of the Study.

Even with two sites, recruiting a sufficient number of participants proved difficult. We initially allocated 12 months for recruitment however even after 30 months we achieved only 48% of the target numbers. A number of factors affected this. Initially, recruitment was limited to those defined as ‘ready to quit’ – based on Prochaska and DiClemente’s model of change [18]. This proved too restrictive and was later expanded to those who expressed any desire to alter their smoking behaviour or reduce consumption. Other factors included: the relative isolation of the BOABS Study teams within the participating ACCHS; the time taken for the local community and health service to develop a sense of ownership of the project; changes to usual referral processes which initially led to the double handling of potential participants by both clinic staff and ARs; and, cultural obligations (see below) which restricted ARs’ access to some community members.

Among the combined intervention and usual care groups, there was a significantly higher overall quit rate (defined as a combination of self report and biologic confirmation with urinary cotinine) [7] in Derby (n = 8/61; 13%) than in Kununurra (n = 3/102; 3%; p = 0.02, χ^2^). The quit rate for a smoking cessation program in Derby prior to BOABS was only 3%. Thus, while a larger number of participants were recruited in Kununurra, the intervention appeared to be more effective in Derby. Factors associated with this greater efficacy included the AR mainly responsible for implementing the intervention at Derby (TK) being more closely engaged with the community long-term and hence viewed as highly credible, more thorough with follow-up, and ‘going the extra mile’ for participants. Evidence of this productive relationship was the fact that 12 months after completion of the study participants were still approaching the AR seeking assistance or reporting on their current smoking status. However, this relationship also likely resulted in some contamination between the usual care and intervention group at the Derby site, and may explain the higher than expected quit rate in the usual care group there (10% actual vs. 3% expected). Also, while there are cultural similarities between the sites, significant historical and social differences between them may have also impacted on the quit rates (Table 1) [19-24].

**Table 1:** Cultural and historical similarities and differences between the two sites

•	After 1880 many Aboriginal people lived and worked on pastoral stations that often encompassed their traditional lands in the Kimberley. The 1968 Federal Pastoral Award, the sudden down turn in Kimberley cattle exports to Britain, and the 300% increase in pastoral lease rent resulted in most Aboriginal people being displaced to towns and missions in the 1970’s [19].
•	Derby was established in 1883 to service the developing pastoral industry. Until recently it was the primary administrative hub for many regional services. Although this growth impacted local Aboriginal people, their resilience and the length of time that has passed, enabled them to adapt, integrate and share in the local economy and other employment opportunities [20].
•	Kununurra was established in 1961 for the damming of the Ord River system. In 1972 the Argyle Dam submerged > 700 km^2^ of Argyle Downs Station, land that is spiritually significant to the Miriuwung and other Aboriginal peoples linked through dreaming tracks to the area. Irrigation farming expanded rapidly to include >200 km^2^ of the Ord River valley [21]. Local Aboriginal people were not consulted about these plans and impacts included dislocation and changes to the natural environment [22,23]. During the 1970s relatively large numbers of former station workers moved into Kununurra where they accessed the Community Development Employment Projects Scheme (a system of welfare) [24].

Focused stakeholder consultation with health care staff and community members highlighted the fact that current tobacco smokers lacked credibility in providing smoking cessation advice. On this basis, the BOABS Study project staff were required to be current non-smokers. CIs and project staff reported this was a strength of the project and reinforced the credibility of the intervention. Unfortunately, after the BOABS Study was completed, the CIs were informed by a credible source that one of the nine ARs employed on the project recommenced smoking whilst still working on the study, potentially reducing that person’s credibility and effectiveness.

In the context of remote Aboriginal health care, the ‘research’ component of the BOABS Study added complexities and barriers that would not have been associated with an integrated smoking cessation program. These complexities included implementing a stand-alone smoking cessation program in two distant locations, requirements for compliance with the Good Clinical Practice (GCP) Australian guidelines [25] and the rigours associated with conducting an RCT more generally. The latter included: minimising contamination; obtaining informed consent and the effect of this on recruitment; extra training for project staff to carry out the research component of the project; and the lack of integration with other clinical services inherent to an RCT in which, whilst allocation is concealed, the intervention cannot be blinded.

Contamination was a potential issue with the BOABS Study with individuals being randomised within individual health services − although the stand-alone nature of the program, separate to routine clinical care, minimised this. Because the BOABS Study was conducted in relative isolation from local clinical services there was sometimes confused delineation between the role of the study and usual clinical care. For example clients were occasionally referred to the study for assessment and provision of NRT despite this remaining a responsibility of the local health services. To minimise such contamination, it was necessary to provide repeated training to both clinic and project staff regarding their roles and responsibilities.

Conducting the Study in a remote location such as the Kimberley also had important implications for staff recruitment, retention and training. Over the five year life of the project (mid-2008 to mid-2013) the BOABS Study had three years funding for a 0.8 full-time equivalent (FTE) project manager and two FTE ARs.

Difficulties were encountered in recruiting and retaining an experienced project manager. Over the first 29 months of the study the position was filled by four individuals (two in Derby and two in Broome) − after which one of the CIs (JM, based in Broome) had to take over management of the project. Similarly, the AR positions were filled by nine individuals. AR turnover was more frequent in Kununurra and, after the last AR there resigned, the main AR from Derby (TK) had to complete participant follow-up in both sites. A related issue was high AR absentee rates due to illness of self or family members and other cultural obligations, which had a similar impact on availability of staff. Such family and cultural commitments reflect the realities of the lives of local Aboriginal people. For programs to be implemented within a framework of cultural safety staffing, and funding of clinical research and programs need to provide sufficient redundancy to ensure program continuity in the face of such absences. Unfortunately available funding meant that we could not recruit additional staff to fill these gaps in the BOABS Study.

It was important to have local ARs who could identify and respond to cultural issues. Amongst the factors that the ARs considered were: managing issues arising from inter-gender relationships (see Table 2 for issues relating to ‘jealousy’); frequent funerals (‘sorry business’) which are an inevitable consequence of both the higher death rate and the larger families of Kimberley Aboriginal people; family issues (conflicts with in-laws, family feuding); observance of cultural obligations and ceremonies; appropriate times for visiting; and appropriate protocols for approaching community members. These factors had an impact on who could be approached, when they could be approached and whether a chaperone was required. These issues were common to both sites, with some local variation, but recognition and local solutions, brokered by ARs, minimised the potential for problems to arise between researchers and participants, strengthening the framework of cultural safety for the BOABS Study.

**Table 2:** Reflection of the main Aboriginal BOABS Study researcher in Derby on dealing with participants in jealous relationships

	*Challenge*
•	Some times when I am working alongside with male or female participants I can pick up if there is jealousy amongst the partners. I need to follow up participants weekly, monthly, group session invitations, and for 6 and 12 month check-ups.
	*Solutions*
•	To respect them you have to try and work with the both of them equally together. This can mean having both present during sessions.
•	I looked up the English dictionary, ‘jealousy’ (noun): ‘Unhappy feeling that someone you love loves someone. A feeling of being unhappy and upset because you think someone who you love is attracted to someone else.’
•	All races experience jealousy. When I am working with countrymen [Aboriginal people] I know my place in my own relationship and so I know how not to cross boundaries with participants.
	*What does this mean for the BOABS Study?*
1.	Being of Aboriginal descent, you kind of know about jealousy.
2.	If you (as a woman) call a male participant’s mobile/house phone you talk to the female first; explain who you are and what you do, so they know.
3.	If you are working with a male; you do the same. If the partner doesn’t like it they will tell you to your face, but most time they are okay with it.
4.	Sometimes you will sign someone up and their partner is non-Aboriginal (white person). They like to join with their partner to give up smoking. I tell them they can join up but not with the BOABS Study. He or she can go through the clinic system to get their NRT [nicotine replacement therapy] and they can still support each other.
•	If you work alongside with your BOABS participant you have a better working relationship with them.
•	And some time if you want to achieve something you just have to do a little bit extra to get there, like respecting their partner’s concerns.
•	You will be fine, they will work with you.

One of the aims of the BOABS Study was to increase the research capacity of local Aboriginal people and organisations. The ARs brought cultural expertise and brokerage skills which were essential for the success of the study, and these were supplemented by providing them with training in both the delivery of smoking cessation support and conducting a research project. Training included initial workplace and project orientation, and focused education and training relating to counselling and motivational interviewing, utilisation of existing smoking cessation training resources (e.g., Fresh Start [26]), and orientation to NRT protocols and use. These structured education and training opportunities were supplemented with practical day-to-day guidance, support and workplace-based updates and consolidation with a particular emphasis on early support following initial employment.

The project focused on local delivery of education and training for staff that reflected the realities of the local environment − rather than sending them to distant, often city-based, locations for training. However, it was often difficult to identify appropriate training providers in our remote location. Furthermore, the high turnover of staff meant that there was high demand for training and this placed a greater than expected burden on resources.

Competing health service priorities were an additional issue, particularly as ARs were sometimes recruited from a relatively finite pool of existing local clinical service staff. While employment by the BOABS Study provided an additional career pathway and training, it often represented part-time employment for ARs who had other responsibilities to the health service. The ARs were at times redeployed to undertake more urgent health service duties when local staff shortages occurred. We attempted to address this by recruiting ARs from outside the services so they would be less likely to be co-opted into other roles. This led to improved recruitment effectiveness in Kununurra but also to more challenges in providing supervision from Broome which probably contributed to a less thorough intervention. Whilst ARs required an understanding of and linkages with the local health services they did not necessarily require clinical qualifications to fulfil this role.

Although conducting an RCT in this setting was difficult, there were positive elements. For example, ongoing evaluation was built into the project and this had two important consequences. The first was continuous improvement of the project, including refinement of the research protocol. Second, regular feedback and consultation with local health services strengthened their engagement and was important in ensuring ongoing commitment and support for the project and smoking cessation initiatives more generally.

## Discussion

The BOABS Study was challenging, though worthwhile, and lessons learnt from our experience are likely to be valuable in informing the design of future research projects, smoking cessation interventions and programs more broadly. First, although RCTs are generally considered the ‘gold standard’ in research, the results of this project highlight some of the difficulties with RCTs and support the argument that in complex public health interventions [27] and clinical individualised interventions such as BOABS, other methodologies may well be better suited to the aims. For example individual randomisation meant that the project could not test the effectiveness of operational changes to clinical service provision as these would affect intervention and control participants [24].

Alternative methodologies to RCTs that could work in remote health services include stepped-wedge and cross-over trials, and plausibility and adequacy evaluations [24,28]. In stepped wedge and cross-over trials all health services receive the intervention, but in random order. The intervention effect is estimated by the between-cluster (those awaiting the intervention and those receiving the intervention) and within-cluster (before and after) comparisons. Plausibility evaluations use an observational design with a comparison group, while in adequacy evaluations process indicators and outcome data are used to suggest if the intervention is having an important effect.

The results of the BOABS Study also demonstrated the importance of local Aboriginal ownership, commitment, participation and control. This included knowledge of local communities, the flexibility to adapt interventions to local communities and circumstances, and taking sufficient time to allow this to occur. Short-term project funding undermines community control and ownership, and program development and delivery [29,30]. Researchers, including ourselves, can also be optimistic and/or naïve in anticipating unrealistic timeframes for completing research projects in settings characterised by significant structural barriers and competing community priorities [31]. The different cultural and historical background to the sites may have impacted on the relative availability of appropriate researchers and different quit rates observed. It is also likely that the motivation of ARs played an important role in BOABS Study participants quitting smoking. Successful programs will need to strive to develop and maintain such motivation.

A possible limitation of this study is that members of the research team were involved in coding their own notes. We attempted to mitigate this by group discussion and by taking a consensus approach to coding. Another limitation was that limited detail was recorded by ARs in their diaries at the start of the project.

Stand-alone programs in primary health care which are not integrated into core services encourage ‘siloing’ of the workforce [32]. Conducting the BOABS Study as an RCT meant that to some extent it was separate from broader clinical activity and despite our efforts was similar to a stand-alone program. This resulted in problems that could be overcome by integration of the intervention into usual primary health care practice. This is something that applies to many stand-alone interventions.

Vertical programs that require a rapid response (e.g., H1N1 vaccinations for swine flu) do have a place for discrete time-specific intervention [33] or for conditions of low prevalence that require specialised staff [34] (e.g., rheumatic heart disease). However, vertical programs are less likely to be sustainable in the long-term [30]. Where conditions are common they are generally managed in primary care settings which means programs to address them need to be integrated into usual existing primary health care practice [35].

Our study suggests a number of ways in which programs can be integrated (see Table 3 for a framework) [36]. These include high quality program planning; organisational policies that support the program; provision of adequate resources; valuing the program; workforce planning and role clarity; normalisation of activities; automatic routine data collection; and shared integrated information systems for program planning, evaluation and improved patient care.

**Table 3:** Synthesis of what is required to conduct a successful integrated clinic based smoking cessation program in Aboriginal Community Controlled Health Services

•	Rationale: worth investing in more than other programs as it is self-sustaining [36]
•	Need significant level of support from governance structure and senior staff
•	At least medium term funding (e.g., 5 years)
•	Responsive to community needs and priorities
•	Health service operations
	▪ At least one person dedicated to smoking cessation ‘Program Driver’
	▪ Environment – e.g., appropriate visual reminders in clinic for staff and patients
	▪ Clinic routine – document status and extent of smoking
	▪ Reasonable data collection requirements & frequent feedback highlighted at regular clinic meetings
	▪ Clear protocols for program: clinic operations; medication management; and ongoing support for prospective and recent quitters
•	Role clarification for staff
	▪ All staff (both non-smokers and smokers) have practical training to have a brief discussion of smoking and provide appropriate encouragement and support (locally targeted brief intervention)
	▪ Several clinic staff trained as expert quit smoking workers (balance of gender, seniority, cultural considerations)

## Conclusions and implications

The keys to the success of this intervention were: local development, ownership and participation; worker training and support; and working within the framework of cultural safety. Main barriers were limited time and resources and the methodological requirements of RCTs to be separate and not integrated into day to day services. Interventions targeted at particular health problems are best integrated with usual primary health care. Research to investigate complex interventions in Indigenous health should not be limited to randomised clinical trials and funding needs to reflect the additional, but necessary, cost of providing for local control of planning, and implementation.

## Abbreviations

ACCHS: Aboriginal Community Controlled Health Service; AR: Aboriginal Researcher; BOABS: Be Our Ally, Beat Smoking; BRAMS: Broome Regional Aboriginal Medical Service; CI: chief investigator; DAHS: Derby Aboriginal Health Service; FTE: Full-time equivalent; NRT: Nicotine replacement therapy; OVAHS: Ord Valley Aboriginal Health Service; RCT: Randomised controlled trial.

## Competing interests

The authors declare that they have no competing interests.

## Authors’ contributions

All of the authors made contributions to the design and analysis of the ‘BOABS Study’. JM is an investigator and managed the project from September 2010 until it was completed. TK is an Aboriginal Researcher and was the main researcher at DAHS. JM and TK were responsible for data collection. JM and DA provided regular support to ARs delivering the intervention. DG led the analysis of the qualitative data. JM, TK, DA, SM and DG attended at least two workshops where the qualitative data was analysed and interpreted, and the manuscript was drafted. All authors contributed to the interpretation of the findings and provided critical review of the manuscript. All authors read and approved the final manuscript.

## Pre-publication history

The pre-publication history for this paper can be accessed here:

http://www.biomedcentral.com/1471-2458/14/579/prepub
